# Interaction between albumin originating from persons with uncontrolled diabetes mellitus type 2 and food antioxidants

**DOI:** 10.5599/admet.2892

**Published:** 2025-09-29

**Authors:** Miloš Šunderić, Dragana Dekanski, Olgica Nedić

**Affiliations:** Institute for the Application of Nuclear Energy, University of Belgrade, Belgrade, Republic of Serbia

**Keywords:** (Dihydro)lipoic acid, glucose metabolism, oleuropein, resveratrol, transport/activity of antioxidants

## Abstract

**Background and Purpose:**

Dietary interventions are a cornerstone in the management of type 2 diabetes mellitus. The efficiency, however, depends on pharmacokinetic factors, including the interaction of food ingredients with plasma proteins. The concept of this study was to investigate the binding effects of three pronounced antioxidants present in the Mediterranean diet: resveratrol, (dihydro)lipoic acid and oleuropein, with albumin isolated from persons with diabetes (HbA1c 63±7 mmol/mol, or 7.9±0.6%) and healthy persons, carrying its intrinsic ligands.

**Experimental approach:**

Spectrofluorometric analysis, native electrophoresis and immunoblotting were performed with albumin before and after the interaction with antioxidants.

**Key results:**

Fluorescence spectra of the protein from two study groups were similar, whereas a spectrum of methylglyoxal-modified albumin (*in vitro* oxidised) was different. Calculated binding constants were also similar for the two study groups for all three ligands. Kinetic fluorescence measurements revealed significantly altered activity of albumin-bound (dihydro)lipoic acid in persons with diabetes compared to healthy individuals, and no significant difference in the activity of resveratrol in expressing antioxidant protection of albumin upon its exposure to oxidative stress.

**Conclusions:**

Although the findings should be further validated using other antioxidants and glycated albumin derived from persons stratified according to the severity of a disease, the results have documented that *in vitro* methylglyoxal-oxidised albumin, routinely employed for diabetes-simulated investigations, was shown not to reflect this pathophysiological condition properly and not to be adequate for the assessment of relevant nutritional/biochemical potential of food antioxidants.

## Introduction

Diabetes mellitus is a medical condition characterised by disturbances in metabolism, leading to long-term complications. Since 10.5% of the adult population, which is approximately 537 million people, are diagnosed with diabetes, and the prevalence is expected to increase to 643 million by 2030 and 783 million by 2045 [1], diabetes has emerged as a significant global public health concern and investigations related to this disease are always actual, both in respect to its etiology and consequences. Elevated plasma glucose levels have been shown to induce the generation of free radicals and the accumulation of oxidation products [2]. When the intracellular antioxidant system is unable to counterbalance an increased oxidative stress, stress-sensitive intracellular signalling pathways are activated, leading to cellular damage, development and progression of various diseases, including diabetes mellitus type 2 [3].

Glycooxidation (glycation) is a specific type of structural modification accompanying diabetes, initiated by glucose metabolic products glyoxylic acid, pyruvic acid and methylglyoxal (MGO). Albumin, a unique carrier molecule of a number of substances in blood, is prone to it. Glycation of albumin may affect its conformation, motion of domains, reactivity of Cys34-thiol group and interaction with ligands [4] and assumes formation of a heterogeneous group of end products [5]. There are several glycation sites on albumin; some of them have been suggested to involve ligand-binding sites [6].

Previously reported studies on the interaction between glycated albumin and ligands were performed mostly after *in vitro* modification of this protein, applying MGO, sometimes also after defatting albumin prior to oxidation. Although drastic conditions are often necessary to capture the effect and investigate its mechanism, in vivo pathophysiological events are not necessarily as strong, which was the rationale behind this study. The concept was to investigate the binding effects of three pronounced antioxidants present in the Mediterranean diet: resveratrol (most abundant in grapes), (dihydro)lipoic acid (DHLA, leafy vegetables), and oleuropein (olives), with albumin isolated from diagnosed persons with diabetes and uncontrolled glycaemia, carrying its intrinsic ligands.

Antioxidants scavenge free radicals, chelate redox-active metal ions, serve as cofactors of antioxidant enzymes, activate gene expression and/or protect their binding partners from an oxidative attack. Dietary interventions are a cornerstone in the management of diabetes and the Mediterranean diet was shown to exert beneficial health effects [7]. However, the efficiency of food antioxidants is highly dependent on various pharmacokinetic factors, including binding to plasma proteins. The aim of this study was to investigate the binding of the chosen antioxidants to albumin glycooxidised under pathophysiological, rather than artificial, conditions, to assess a more natural situation.

## Experimental

### Sample collection

Blood samples were obtained from persons with type 2 diabetes (*n* = 20, 13 men, 7 women, age 44-72 years) who underwent regular laboratory control in respect to glucose metabolism in the Institute for the Application of Nuclear Energy (INEP), which is included in the medical health service in the Republic of Serbia. A control group consisted of healthy individuals, H, who underwent regular examinations (*n* = 20; 10 men, 10 women, aged 40-66 years). Serum samples remaining after the requested analysis were used for the isolation of albumin, with the informed consent of participants and in accordance with the Approval of the Ethical Committee of INEP (Approval no. 0203-07-013/010/2025). The concentrations of glucose and HbA1c are given in Table 1. Serum samples from each group were grouped into two pools, resulting in four pooled samples: DM1, DM2, H1 and H2. Pools (in duplicate) were prepared by mixing 0.1 mL of 10 serum samples from the same group, with each serum used only once in a pool.

**Table 1.** Data on concentrations and binding constants for two study groups (DM and H)

	DM group	H group	*p*-value
Glucose concentration (SD), mM	11.1 (1.5)	5.3 (0.3)	<0.001
HbA1c concentration (SD), mmol/mol HbA1c concentration (SD), %	63(7) 7.9 (0.6)	32(3) 5.1 (0.3)	<0.001
Total protein concentration in the preparation of isolated albumin, g L^-1^	DM1 pool: 37.2 DM2 pool: 28.4	H1 pool: 32.0 H2 pool: 36.2	
Albumin concentration in the preparation of isolated albumin, g L^-1^	DM1 pool: 36.9 DM2 pool: 28.0	H1 pool: 31.3 H2 pool: 35.5	
Purity of the isolated albumin, %	99	98	
*K*_a_ for resveratrol (SD), M^-1^	2.0 (0.03)×10^4^	2.4 (0.06)×10^4^	
*K*_a_ for DHLA (SD), M^-1^	2.6 (0.22)×10^3^	3.2 (0.25)×10^3^	
*K*_a_ for oleuropein (SD), M^-1^	3.2 (0.14)×10^2^	2.3 (0.12)×10^2^	

### Isolation of albumin from serum

Albumin was isolated from pooled samples using the procedure described by Jovanović *et al.* [8]. A saturated ammonium sulphate (SAS) solution was added to 1 mL of serum, with constant mild mixing, until the solution reached 54 % saturation. A precipitate was removed by centrifugation (10000 *g* for 10 min) and SAS was added to the supernatant to reach 70 % AS saturation. After incubation at 4 °C for 45 min, the mixture was centrifuged, the precipitate dissolved in 1 mL of 0.05 M phosphate buffer pH 7.4 (PB) and the solution subjected to repeated filtration using a centrifugal filter device with 30 kDa cut-off (Amicon, Merck KGaA, Darmstadt, Germany) and PB to remove AS. The final volume of albumin solution was adjusted to 1 mL with PB and the protein concentration was determined using both Biuret (total proteins) and Bromocresol green (albumin) reagents (BioSystems, Barcelona, Spain). The purity of albumin was tested by native electrophoresis using commercial human albumin (Sigma-Aldrich, Burlington, USA) as a standard.

### Modification of albumin by oxidation with MGO

Both albumins isolated from healthy persons and the commercial protein were modified by MGO. The concentration of albumin was adjusted to 0.37 mM using PB and 0.442 μL of 40 % MGO solution (Sigma-Aldrich, Burlington, USA) was added, resulting in a final concentration of 7 mM. A mixture was thoroughly vortexed, left at 37 °C for 24 h and subjected to repeated filtration using a centrifugal filter device with 30 kDa cut-off and PB to remove unreacted MGO.

### Spectrofluorometric analysis of albumin, its interaction with antioxidants and structural stability under oxidative stress

Fluorescence spectra of the commercial, native (isolated) albumin, MGO-modified and antioxidant-bound were recorded using an RF-6000 spectrofluorometer (Shimadzu, Kyoto, Japan). Each measurement was performed in duplicate (for each sample) and the results were presented as an average spectrum for a study group. A wavelength of 295 nm was used to excite the Tryptophan residue, while an emission spectrum was followed up in the range 300 to 550 nm.

The same procedure was applied to examine the effect of albumin interaction with antioxidants. A starting concentration of albumin was 5 μM, whereas concentrations of ligands were adjusted to obtain measurements that could reliably monitor a binding affinity: resveratrol (Carl Roth, Karlsruhe, Germany) range 0 to 10 μM, DHLA (Sigma-Aldrich, Burlington, USA), 0 to 500 μM and oleuropein (Extrasynthese, Genay, France), 0 to 1500 μM. Emission spectra were corrected for the absorption effect of albumin + ligand complex following Equation (1):



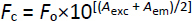

(1)


where *F*_c_ and *F*_o_ are the corrected and observed fluorescence of albumin + ligand complex, while *A*_exc_ and *A*_em_ are the absorbance of the complex at the excitation and emission wavelength, corrected for the absorbance of the ligand alone (as the concentration of ligand increased, it significantly exceeded the concentration of protein and, in the case of resveratrol and oleuropein, contributed to spectra). *F*_c_ was calculated for each albumin + + ligand combination, with respect to the origin of albumin, type of ligand and its concentration.

To calculate a binding constant (*K*_a_), Equation (2) was used and the corresponding graph was plotted:



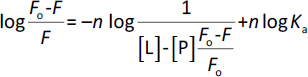

(2)


where *F*_0_ and *F* are the fluorescence intensities of albumin at the maximum in the absence and in the presence of the ligand, [L] and [P] are the concentrations of the ligand and the protein, *K*_a_ is the binding constant and n is the Hill coefficient, *i.e.* an approximation of the number of binding sites. Spectrofluorometry was also applied to compare the relative content of two typical glycooxidative protein modifications in samples: advanced-glycation end-products, AGEs (excitation at 370 nm) and dityrosine, DT (330 nm) [9,10].

To compare two study groups with respect to the potential of bound antioxidants to protect albumin against oxidative stress, a kinetic follow-up of protein structural change, expressed as a change in the intensity of the fluorescence maximum after excitation at 295 nm, was performed. Albumin (5 μM) and ligand (10 μM resveratrol or 500 μM DHLA) were incubated for 30 min at 25 °C, and an oxidizing agent, 2,2'-azobis(2-amidinopropane) dihydrochloride (AAPH) was added to reach 50 mM, a mixture was thoroughly mixed, and the fluorescence spectra were recorded for the next 10 min, at 1-min intervals. Structural change was detected as a reduction in fluorescence at the maximum rate per minute, for each measured time point (Δ*F* / min^-1^).

### Native electrophoresis and immunoblotting of albumin

Standard native PAGE was performed on 10 % gels. The following samples were examined: commercial and isolated albumin (DM and H), albumin plus antioxidant, albumin plus AAPH, and albumin plus antioxidant plus AAPH. Gel staining was achieved using Coomassie brilliant blue (CBB). Proteins were transferred to NC membrane and immunoblotting was performed using rabbit anti-human albumin antibody (Sigma-Aldrich, Burlington, USA), HRP-conjugated secondary antibody and ECL substrate (Thermo Fisher Sci, Norristown, USA). Gels and membranes were scanned using the ChemiDoc MP Imaging System (Bio-Rad, Hercules, USA).

### Statistical analysis

Numerical data for two study groups were compared using a two-sample *t*-test.

## Results

**Figure 1.**
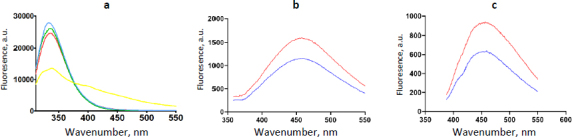
Fluorescence spectra of albumin (5 μM) originating from H (blue line), DM (red line), commercial (green line) and MGO-modified (yellow line) samples after excitation at 295 nm (a – Tryptophan signal, all samples), 370 nm (b – AGEs, albumin samples isolated from serum) and 330 nm (c – DT, albumin samples isolated from serum)

The purity of the isolated albumin was tested by measuring and comparing the total protein and albumin concentrations in the preparations (Table 1), as well as by electrophoresis. The isolated albumin was 98 to 99 % pure. Average fluorescence spectra of albumin from two study groups were similar, whereas a spectrum of MGO-modified albumin was different, both in the shape and intensity, but not in the position of the maximum (Figure 1a). These results indicate that albumin molecules originating from individuals with diabetes are quite distinct from those subjected to MGO *in vitro*, under conditions similar to those reported by other researchers [5,11,12], justifying our concept of examining albumin modified *in vivo*. Examination of the relative content of AGEs and DT has shown higher levels of both modifications in albumin isolated from persons with diabetes than from healthy individuals (Figure 1b-1c).

**Figure 2.**
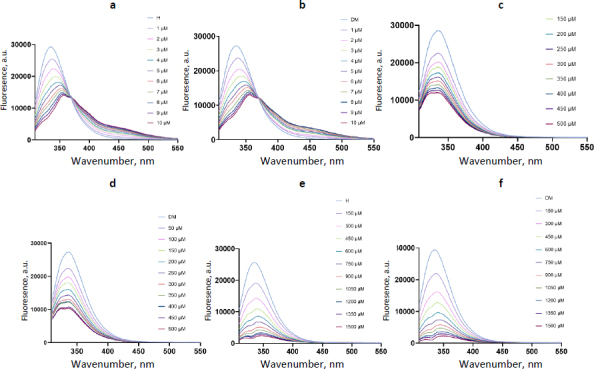
Fluorescence spectra of albumin originating from H and DM samples after interaction with ligands: resveratrol (a and b), DHLA (c and d) and oleuropein (e and f).

Fluorescence spectra of the isolated albumin subjected to interaction with antioxidants showed a characteristic quenching effect (Figure 2a-2f). Interaction of albumin from either group of samples with resveratrol caused a spectral shift not seen with the other two ligands, suggesting a more pronounced effect on the complex structure. The Hill model was chosen for the calculation of binding constants, as one of the proposed models for albumin-ligand interactions, without considering alternative models. *K*_a_ calculation was based on a single-wavelength analysis, which corresponded to the maximum wavelength for each albumin-ligand combination (fitting plots are provided as the Supplementary material). Calculated binding constants were similar for the two study groups for all three ligands (Table 1). A number of binding sites was estimated as 1 for all combinations. *K*_a_ in the case of resveratrol binding can be considered as moderate, in the case of DHLA small, while in the case of oleuropein, it seems that an interaction may not be reliably considered as binding, disqualifying oleuropein from further experiments.

**Figure 3.**
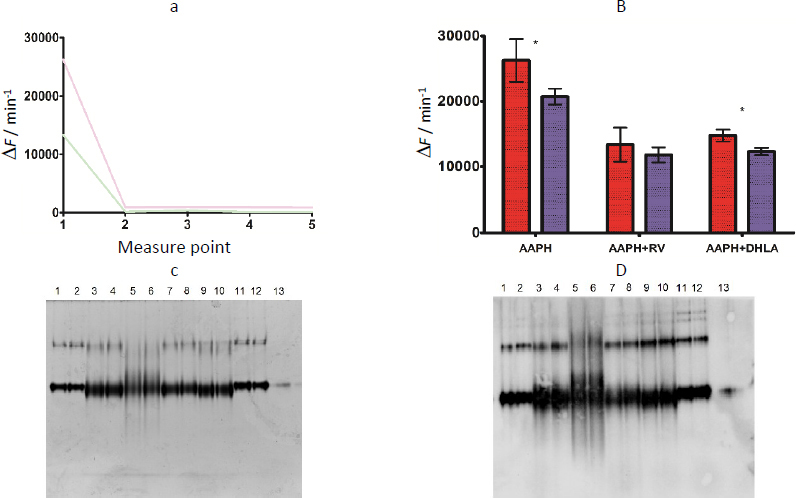
(a) Fluorescence change of albumin originating from H group after oxidative attack by AAPH, in the absence (pink line) and in the presence (green line) of resveratrol - each measurement point defines a change (Δ*F*) that occurred during a 1-min interval. (b) Comparison of results for two study groups: H (purple field) and DM (red field); statistically significant difference is marked with asterisk. (c) Native electrophoresis/CBB staining and (d) immunoblotting with anti-albumin antibody of the following samples: isolated albumin (1, 2), albumin/resveratrol complexes (3,4), oxidised albumin with AAPH (5, 6), albumin/resveratrol complexes + AAPH (7, 8), albumin/DHLA complexes + + AAPH (9, 10), albumin/DHLA complexes (11, 12) originating from two study groups: H (odd samples) and DM (even samples) and a commercial albumin (13)

Antioxidant potential of resveratrol and DHLA bound to albumin was examined as the ability of the protein to resist oxidative attack by AAPH. Time-course measurements of the fluorescence (*F*) at spectral maximum were performed at 1 min intervals for 10 min and the change was expressed as Δ*F* min^-1^ for each measurement point. An example of the graphic presentation of the data is given in Figure 3a (for albumin from H group, subjected to AAPH alone and in the presence of resveratrol). As can be seen, the interaction is very fast and terminates within a minute. The area under the graph was used to monitor relative change, as it was directly proportional to it. Albumin from healthy persons, both alone or in complexes with either resveratrol or DHLA, was more resistant to structural change in the presence of AAPH (smaller Δ*F* min^-1^) than albumin from persons with diabetes (Figure 3b). The difference was statistically significant in the case of DHLA and nearly significant in the case of resveratrol.

Samples that remained after kinetic measurements were subjected to native electrophoresis and immuneblotting with anti-albumin antibody (Figure 3c-3d). A major protein species was the albumin monomer, although the dimer was also present. No other proteins were detected upon gel staining. No significant difference was observed between isolated albumin molecules from the two groups (samples 1 and 2), either in terms of protein band shape or immunoreactivity, confirming the similarity of molecules in the H and DM groups during their movement in the electric field or recognition by the antibody. Interaction with resveratrol (samples 3 and 4), but not DHLA (samples 11 and 12), affected albumin in terms of the appearance of additional molecular forms - complexes (signal widening). AAPH alone modified significantly albumin, resulting in a wide protein distribution without a clear band (samples 5 and 6). The interaction of albumin with either resveratrol (samples 7 and 8) or DHLA (samples 9 and 10) reduced molecular scattering when AAPH was present, exerting a protective effect against structural changes and confirming previous spectrofluorometric results. No significant difference was detected between the two groups of samples in this experiment.

## Discussion

Plasma proteins reversibly bind bioactive compounds, both in the native state and their metabolites. Antioxidants are among the most intensively studied phytochemicals; however, their biological activity needs additional examination, having in mind their relatively low bioavailability (especially lipophilic compounds) and metabolic processing after intestinal absorption. On the other hand, plasma proteins modified by diabetes may have altered affinities for antioxidants and oxidants themselves may have altered activity in complexes. For example, the affinity of plasma proteins originating from persons with diabetes for polyphenols was measured to be an order of magnitude lower than of healthy persons [13].

Albumin oxidised *in vitro* by MGO, a common approach to investigate its interaction with ligands, does not accurately reflect the physiological conditions, as shown in our first experiment. The result is not surprising, since albumin is just one of the plasma proteins, MGO is just one oxidative agent, physiological changes occur over a much longer time than experimentally, the physiological concentration of MGO is smaller than in experiments conducted *in vitro* [14], and many other factors participate in the interplay within an organism. Having that in mind, it seemed relevant to examine the binding of antioxidants to albumin isolated from persons with diabetes, altered under pathophysiological terms and loaded with intrinsic ligands. Conditions used to isolate albumin were not harsh (gradual separation employing AS) and have already been confirmed as suitable for such a type of examination [8].

A portion of glycated albumin (fructosamine) in healthy persons is 6-13 %, whereas it can reach 30 % in persons with type 2 diabetes [15]. Albumin has nine binding sites for fatty acids (FA), two major drug-binding sites (Sudlow’s sites I and II) and one metal-binding site [16,17]. A degree of oxidation and the reactivity of Cys34 thiol group depend on the type of the bound FA [18], and FA may also modulate the binding of other ligands. A profile of free FA in plasma, and consequently bound to albumin, may differ in persons with diabetes compared to healthy people. The mechanism underlying this change is unclear, but dietary fats and modulation of lipid metabolism are likely contributing factors [19]. Thus, isolated albumin molecules, carrying physiological ligands, more reliably represent a pathophysiological condition of diabetes than do purchased molecules that have been extensively modified *in vitro*.

A choice of antioxidants for this study relied on the recommended foods for the improvement of metabolic disorders: grapes/wine (resveratrol), green vegetables (lipoic acid) and olives/olive oil (oleuropein). The protective/antidiabetic effects of resveratrol involve the inhibition of oxidative stress and inflammation, enhancement of insulin sensitivity, regulation of lipid metabolism, as well as promotion of GLUT4 expression [20-22]. *K*_a_ of the commercial albumin for resveratrol is 2.56×10^5^ M^-1^, there is one binding site in the subdomain IIA and the interaction is based mostly on the formation of hydrogen bonds [17]. Lipoic acid (LA) can be synthesized in humans in small amounts, and its reduced form, dihydrolipoic acid (DHLA), is formed intracellularly. It is suggested that the ratio between LA and DHLA regulates the activity of cell enzymes [23]. DHLA is a strong antioxidant that can ameliorate chronic inflammation [24]. *K*_a_ of the commercial albumin for DHLA is 10^4^ M^-1^ and the preferential binding site is in the subdomain II [25]. Oleuropein can scavenge reactive oxygen and nitrogen species, induce the expression of nitric oxide synthase in cells, prevent oxidation of LDL, and reduce cholesterol in the blood. It also exerts anti-cancer and antimicrobial activity [26]. Literature data on oleuropein binding to albumin express disagreements. He *et al*. identified the pocket of the subdomain IIA as a binding site and calculated *K*_a_ of the order 10^4^ M^-1^ [27]. According to Roche *et al*. [28], on the other hand, oleuropein interaction with albumin did not modify the hydrophobic environment around Tryptophan214, and no clear binding site could be identified. All *K*_a_ values calculated in our study were smaller than those reported previously for commercial albumin (Table 1), which could be related to the presence of already bound ligands. Significant difference in *K*_a_ values between the two groups was not found. When separated albumin glycooxidised forms were compared with non-glycated forms, a 50 % reduction in affinity for bilirubin and unaltered affinity for hemin was measured [29]. A number of studies investigated the binding capacity of glycated albumin, modified either *in vivo* or *in vitro*, and reported increased, decreased or unaltered binding of specific ligands [5].

Albumin is a molecule that undergoes various post-translational modifications associated with diseases. An article by Wu *et al*. [30] reviews albumin modifications and the changes in its function resulting from structural alterations. Authors introduced the concept of “effective albumin concentration” as opposed to the total concentration. Diabetes, liver, cardiovascular, kidney and other diseases induce structural changes, the most common being oxidation, nitration, nitrosylation, glycation, truncation and dimerization [30]. The majority of studies examined either properties of albumin, such as antioxidant activity, or its binding affinity upon modification, most often for drugs, metal ions and FA [31,32]. Some results, reported in different studies, are conflicting. Pathophysiological factors, such as the amino acid residue affected by modification, co-existence of several modifications and/or the severity of a disease, may be a reason for opposing results, but also a methodological approach. In addition, disease-related substances may accumulate in the organism, such as uremic toxins in chronic kidney disease, and bind to albumin with high affinity, reducing its interaction with other ligands [33].

Common to the published studies is the investigation of the amount of bound ligand to the modified albumin, not its activity. It is generally assumed that an increased or decreased quantity of the bound ligand is directly proportional to its activity. This may not be the case if the albumin affinity for ligand is considerably changed or if some ligand modification occurs as well, due to the altered interacting surface of albumin and, consequently, formation of alternative bonds. Thus, evaluation of the ligand activity after its interaction with a carrier protein offers specific information on its physiological potential.

Our diabetes group did not contain extremely severe cases. Soudahome *et al*. [34] reported that persons with HbA1c 10.0±0.5 and 16.0±1.7 % had 1.7- and 3-fold more glycated albumin than those without diabetes. Albumin from the first group had an affinity for liraglutide (an antidiabetic drug) similar to the control albumin, whereas an increased *K*_d_ was measured for albumin from the second group. Our experimental data refer to the entire albumin population and not just glycooxidised, so even in individuals with a greater proportion of modified albumin, this molecular form is a minor species. Both our and literature results support the choice of isolated albumin as a more reliable substrate to study real diabetes-associated events that depend on diabetes stage.

Albumin from persons with diabetes exposed to AAPH suffered from greater structural change than albumin from healthy persons, which could be associated with diabetes-induced modifications such as AGEs and NTs, and possibly the presence of intrinsic ligands. The binding of DHLA to albumin significantly reduced the effect of AAPH, but the protection was greater in the case of albumin from healthy persons. Therefore, although native fluorescence spectra, affinity constants, electrophoretic movement or immunoreactivity of albumin from persons with diabetes and healthy people exhibited no significant difference (or could not be captured due to limited sensitivity of techniques), the activity of DHLA bound to albumin was different. This finding could imply that certain structural changes in albumin due to diabetes may influence the behaviour of a particular bound antioxidant and possibly affect its physiological involvement. Different behaviour of different antioxidants may be attributed to their distinct molecular structures and/or specific binding sites on albumin. However, no conclusions can be made without competitive displacement studies, other experiments and evaluation applying *in silico* modelling.

The interaction between albumin and antioxidants is just one factor that affects their bioavailability. Other factors include chemical structure, food matrix, transformation by host gastrointestinal and microbial enzymes, transport, processing and interactions within the body [35]. Food matrix becomes a crucial factor when antioxidants are physically entrapped in the matrix or associated with other molecules or cell structures [36]. A particular antioxidant may be free in one food and not in another. Furthermore, foods ingested together enable interaction of their components, resulting in an overall bioavailability effect. Food preparation, such as thermal processing or fermentation, further influences both the bioavailability and the chemical form of a certain antioxidant.

Hydrosoluble antioxidants are directly absorbed in enterocytes, while liposoluble form micelles with lipids and bile acids, which then diffuse through the intestinal epithelium or pass linked to transporters. Lipids co-ingested with lipophilic antioxidants increase their emulsification and absorption. Lipophilic antioxidants most often undergo conjugation during the passage through the liver. Excretion of antioxidants and their metabolites occurs *via* the kidneys (urine) or liver (bile/faeces). Even without going into details, it is evident that numerous factors influence the final outcome of an antioxidant intake.

## Conclusions

Investigation of the protein/ligand binding offers specific information that refers to a complex process of ligand absorption, distribution, metabolism, excretion and toxicity (ADMET). There are many biomolecules involved, and albumin is only one of them. Although we have shown that the activity of some antioxidants (and others not) bound to albumin may differ in healthy individuals and those with diabetes, the result cannot be interpreted in isolation and without consideration of the entire metabolic mechanism. A limitation of our study is the small sample size (*n* = 20 for each group) and no validation of the possible effect concerning patient variability on the structure of albumin and the activity of bound antioxidants (*e.g.* grade and duration of diabetes, other comorbidities). Pooled samples were used to capture the effect, which limits the statistical power of the study and provides no information on inter-individual variability. Thus, further examination of individual samples originating from a rigorously stratified population should provide more reliable results on the possible involvement of other biochemical and clinical factors in albumin-antioxidant interaction.

## Supplementary material

Additional data are available at https://pub.iapchem.org/ojs/index.php/admet/article/view/2892, or from the corresponding author on request.


