# Supplementary material for: Interaction between albumin originating from persons with uncontrolled diabetes mellitus type 2 and food antioxidants
Source: ADMET DMPK. 2025 Sep 29;13(5):2892. doi: 10.5599/admet.2892 (PMC12662243; doi:10.5599/admet.2892)
Supplement: Supplementary file 1 [file ADMET-13-2892-S1.pdf]

Open Access : ISSN : 1848-7718

http://www.pub.iapchem.org/ojs/index.php/admet/index

Supplementary material to

Interaction between albumin originating from persons with uncontrolled diabetes mellitus type 2 and food antioxidants

Miloš Šunderić, Dragana Dekanski and Olgica Nedić

Institute for the Application of Nuclear Energy, University of Belgrade, Belgrade, Republic of Serbia

ADMET & DMPK **13(5)** (2025) 2892; https://doi.org/10.5599/admet.2892

Interaction between albumin originating from persons with uncontrolled diabetes mellitus type 2 and food antioxidantsj

Representative examples of fitting plots for the calculation of K_a for albumin complexes are shown in Figure S1.

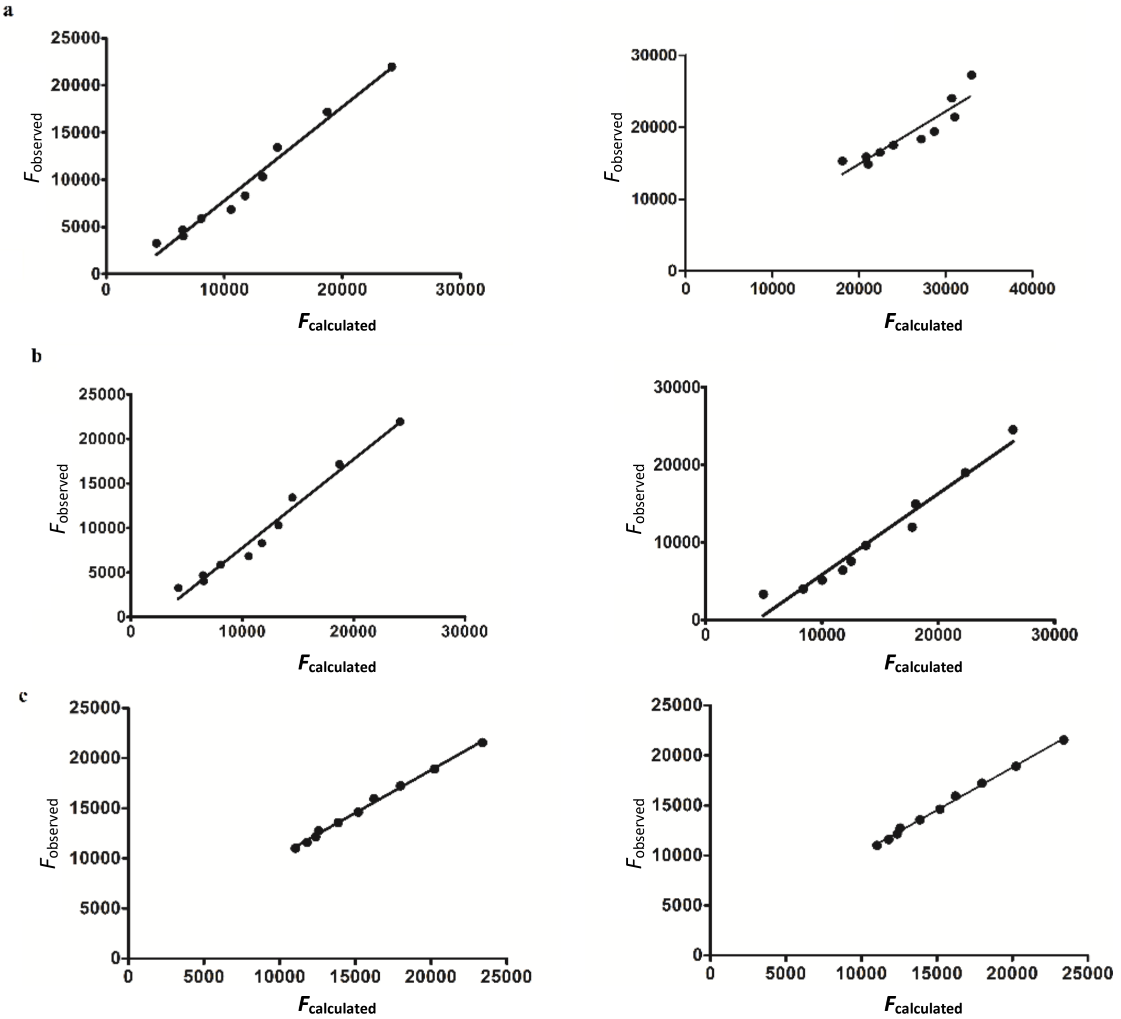

Figure S1. Fitting plots (representative examples) for the calculation of K_a for albumin complexes with resveratrol (a), oleuropein (b) and DHLA (c); albumin samples on the left are from healthy persons and on the right from patients with diabetes
